# Role of Circadian Rhythms in Major Plant Metabolic and Signaling Pathways

**DOI:** 10.3389/fpls.2022.836244

**Published:** 2022-04-06

**Authors:** Ajila Venkat, Sowbiya Muneer

**Affiliations:** ^1^Horticulture and Molecular Physiology Lab, School of Agricultural Innovations and Advanced Learning, Vellore Institute of Technology, Vellore, India; ^2^School of Biosciences and Technology, Vellore Institute of Technology, Vellore, India

**Keywords:** circadian rhythms, carbon metabolism, gene regulatory pathways, plant metabolism, signaling pathways, photosynthesis, flowering and senescence, defense response

## Abstract

Plants require an endogenous regulatory network and mechanism to cope with diurnal environmental changes and compensate for their sessile nature. Plants use the circadian clock to anticipate diurnal changes. Circadian rhythm predicts a 24-h cycle with 16 h of light and 8 h of darkness in response to abiotic and biotic factors as well as the appropriate temperature. For a plant’s fitness, proper growth, and development, these rhythms synchronize the diurnal photoperiodic changes. Input pathway, central oscillator, and output pathway are the three components that make up the endogenous clock. There are also transcriptional and translational feedback loops (TTFLs) in the clock, which are dependent on the results of gene expression. Several physiological processes, such as stress acclimatization, hormone signaling, morphogenesis, carbon metabolism, and defense response, are currently being investigated for their interactions with the circadian clock using phenotypic, genomic, and metabolic studies. This review examines the role of circadian rhythms in the regulation of plant metabolic pathways, such as photosynthesis and carbon metabolism, as well as developmental and degenerative processes, such as flowering and senescence. Furthermore, we summarized signaling pathways related to circadian rhythms, such as defense response and gene regulatory pathways.

## Introduction

Living organisms, such as animals, cyanobacteria, and plants, have an internal endogenous oscillator known as the circadian clock, which predicts the alteration during the light and dark cycles. The term “circadian” was first coined in 1959 by Franz Halberg, originated from two different Latin words “circa” means “around” and “diem or dies” means “day” (McClung, 2006; Srivastava et al., 2019; Man et al., 2020). It permits organisms to synchronize and assemble for daily or seasonal changes depending on the surrounding environmental conditions. The circadian clock is an internal biological timekeeper that helps the plants to attain fitness, proper growth, and development (Inoue et al., 2017). This endogenous oscillator is generally comprised of three different modules: (i) input pathway, which gives information about the surrounding environment; (ii) central oscillator that consists of “canonical clock gene,” which composes the elite clock design; and (iii) the output pathway that constitutes the clock-driven downstream processes. The central oscillator includes complex TTFLs (de Dios et al., 2018) that blend with the post-transcriptional and post-translational modifications (Dalchau et al., 2011; Yan et al., 2021). The circadian clock has a self-reliant mechanism and their metabolic processes can also be administered by circadian rhythm, which was previously studied with the model plant *Arabidopsis*, also in the potato, and rice crops (Kim et al., 2017; Inoue et al., 2018).

The circadian rhythm oscillates daily for about 24 h in the period of light and dark cycles in response to biotic and abiotic factors (Kiełbowicz-Matuk et al., 2014). From the studies of Harmer (2009), it was detected that there are observed three feedback loops, such as morning, central, and evening loops, from the central oscillator, in the model organism *Arabidopsis thaliana*. The central loop consists of CIRCADIAN CLOCK ASSOCIATED 1 (CCA1) and LATE ELONGATED HYPOCOTYL (LHY) (Kim et al., 2016) encode MYB-related transcription factors that are classified as the morning expressed genes. CCA1 modulates clock-independent and clock-dependent responses (Lei et al., 2019). *TIMING OF CAB EXPRESSION* (*TOC1*) is an evening expressed gene from the core or central loop, which is from the family of Pseudo-Response Regulator (PRR) (Shogo et al., 2008). Thus, these core loops combine with the morning and evening loops and help in the primary construction of circadian rhythm in plants (Wang and Ma, 2013).

**FIGURE 1:**
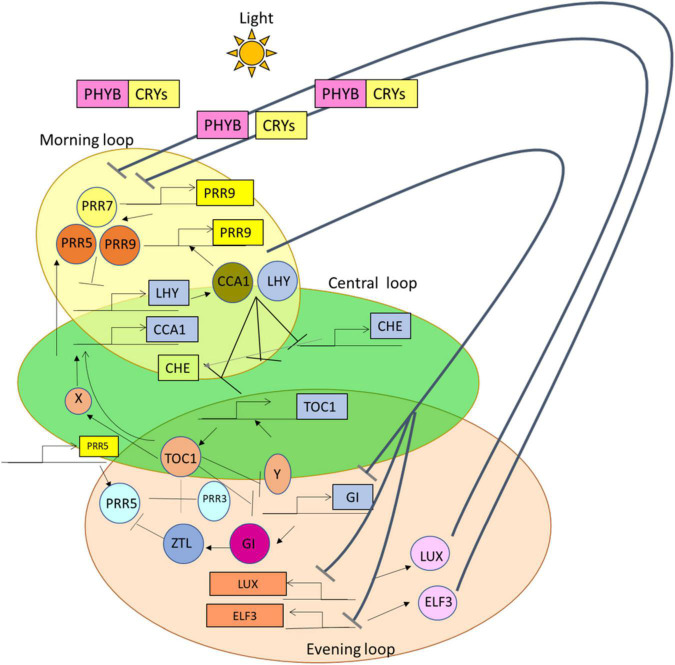
A simplified representation of the suppression of genes having the proteins and photoreceptors present during the functioning of 24 h circadian rhythm. In the presence of light, these photoreceptors Cryptochromes (CRYs) and Phytochrome B (PHY B), which are represented in yellow and pink squares, help in the functioning of genes and proteins; along with the formation of two different negative loops. That is, these morning loop genes [CIRCADIAN CLOCK ASSOCIATED 1 (*CCA1*) or LATE ELONGATED HYPOCOTYL (*LHY*)] combine with Pseudo-Response Regulators (*PRR7* or *PRR9*) and suppress the action of CCA1 or LHY (represented with black lines with arrows). On the other hand, *TIMING OF CAB EXPRESSION* (TOC1) combines with GI, which then leads to the activation of the *TOC1* gene. ZEITLUPE (ZTL) is activated with the help of GI, and then GI itself combines with ZTL and suppresses the function of TOC1 (indicated with the black lines along with arrows).

From the previous studies, it was perceived that in the morning loop, *CCA1* or *LHY* gene forms a negative feedback loop by combining with a PRR7 or PRR9 and represses the expression of CCA1 and LHY (Farré et al., 2005; Nakamichi et al., 2010). Likewise, in the evening loop, the *TOC1* gene forms a negative feedback loop along with GIGANTEA (GI); also it represses an unknown Y factor, which further activates the expression of the *TOC1* gene (Huq et al., 2000; Mizoguchi et al., 2005; Chen et al., 2013; Saini et al., 2019). GI activates ZEITLUPE (ZLT) protein and then acts along with it and targets TOC1 protein for degradation (Figure 1). In addition, *LUX ARRHYTHMO* (*LUX*) is a gene and EARLY FLOWERING (ELF), such as ELF 3 and ELF 4, that is the protein of the evening complex (EC), which forms various sets of interlocked negative feedback loops (Nohales and Kay, 2016). In the previous studies (Rawat et al., 2011; Rugnone et al., 2013; Wu et al., 2016), it is mentioned that *REVEILLE* (*RVE*) genes, *LIGHT-REGULATED WD* (*LWD*) *1* and *2* genes (transcriptional coactivators) (McClung, 2019), and *NIGHT LIGHT-INDUCIBLE AND CLOCK-REGULATED* (*LNK*) genes were the positive regulators found in the loops of the circadian oscillator.

In model organism *A. thaliana*, it was found that circadian cycle duration can be extended or lengthened significantly using the overexpressed transcription factors BBX18, BBX19, and BBX32 (B-Box); also BBX18 increases the speed of this biological cycle and BBX32 overexpression may even lead to late flowering (Yuan et al., 2021). Circadian gating is the process where the circadian rhythm balances the plant response with different environmental cues; thus, the response depends upon the daytime (Belbin et al., 2019). The regulation of the circadian clock is intrinsically connected with the responses toward decreasing in temperature. Adaptation of cold has proteins involved, such as C-REPEAT/DRE BINDING FACTOR (CBF), which has a role as a key regulator (Panter et al., 2019).

The input pathway reveals to extend some of the environmental variations or signals, recognized by the photoreceptors, such as Phytochrome B (PHY B) and Cryptochromes (CRYs), which develops the components of the negative loops and temperature to entrain the stage and waveform of the circadian oscillator (Aguilar-Arnal and Sassone-Corsi, 2015). The output pathway controls several processes, such as reproductive development, hormonal production, defense responses, and the minimal percent of expression of the genome (Shor et al., 2017). Various physiological processes take place, such as stress acclimatization, hormone signaling, morphogenesis, carbon metabolism, and defense response including phenotypic, genomic, and metabolic studies in the later stages, along with the interaction with this circadian clock (Sanchez et al., 2011).

Circadian research has been extremely interdisciplinary, attracting researchers from a wide range of scientific disciplines. The existence of circadian rhythms was demonstrated by astronomer Jean Jacques d’Ortous deMairan in 1729. Jean Jacques d’Ortous deMairan discovered that the daily leaf movements of the heliotrope plant, *Mimosa pudica*, continued in complete darkness that implies the existence of an endogenous time-generating mechanism in accordance with geophysical time. Bünning, however, provided the first evidence for a genetic basis of circadian rhythms two centuries later. Bünning reported that in common beans, the offspring’s period lengths ranged between the extremes of the parent generation’s period lengths.

Circadian rhythms generate a biological time of day measurement. Circadian regulation is an important adaptation in plants to their changing environment. The majority of our understanding of the molecular aspects of circadian regulation in plants comes from controlled laboratory experiments. However, it is becoming clear that the circadian clock plays complex roles in transcriptome coordination under natural conditions, in both naturally occurring plant populations and crop species. This review has a work scheme based on the role of circadian rhythms in the regulation of plant metabolic pathways, such as photosynthesis and carbon metabolism, the regulation of developmental processes, such as flowering and degenerative processes, i.e., senescence, the regulation of plant signaling pathways, such as defense response, and the gene regulatory pathways interrelated with the circadian rhythms. Further, the review will also be highlighted with the research gaps that identify new domains and suggest recommendations for future investigation (Table 1).

**TABLE 1:** Specific genes and proteins present in plant circadian rhythm and its major functions.

S. no.	Names of the genes or proteins	Major functions	References
1.	*CCA1/LHY*	It is induced by the light; also they trigger the genes managed by the clock that is expressed fast in the daytime. Whereas, suppressing genes are expressed in the nighttime.	Green and Elaine, 2002
2.	CRY	It is a blue light-dependent photoreceptor, which has a major role in the development and growth of plants. It helps in promoting flowering duration in plants; mainly it induces hypocotyl growth.	Lopez et al., 2021
3.	PHY	It mainly helps in controlling the development of plants, from the seed germination step to the flowering stage. It is a red light-dependent photoreceptor, which interacts directly with *ELF 3*. Also, in circadian rhythm, it has an essential role in the Red (R) light-mediated entrainment.	Yeom et al., 2014
4.	GI	It has a major role in the flowering pathway; mainly it helps in regulating the circadian rhythm and also the flowering stage. GI acts in between the circadian rhythm and CO, where CO stimulates the flowering by increasing the mRNA abundance of CO protein and *FT* gene.	Mizoguchi et al., 2005
5.	PRR 9/PRR 7/PRR 5	These are triple alleles of PRR, and it has a crucial role in the formation of either positive or negative loop in plant circadian rhythm. Along with their severe phenotype, the photoperiod-dependent flowering can be noticed which can even be the late flowering stage. PIF (Phytochrome Interacting Factors) activity gets inhibited with the help of *PRR 5* and *PRR 7*, which could further result in the lodging of plants.	Nakamichi et al., 2005, 2010; Franklin, 2020
6.	*ELF 3*	*ELF 3* gene has its main role in the input pathway, along with light. It also sends an altered signal about the sensitivity of the central oscillator to the light at a specific time during the circadian cycle.	Hicks et al., 2001
7.	*ELF 4*	Generally, these *ELF* genes have an important role during the flowering stage in various light conditions in the presence of circadian rhythm. It is crucial for the sustainability of rhythmicity under certain constant conditions and the entrainment to the environmental cycle.	McWatters et al., 2007; Fukuda et al., 2020
8.	*LUX ARRHYTHMO (LUX or PHYTOCLOCK1)*	These *LUX* genes along with *ELF* genes has a vital role in the maintenance of circadian rhythm and also in adjusting the development and growth of plants	Liew et al., 2017; Zhang C. et al., 2019
9.	ZTL	It is an important E3 ligase that helps in maintaining proper periodicity during circadian rhythm.	Feke et al., 2021
10.	*TOC 1*	It is present in controlling the elongation of hypocotyl growth which is phytochrome dependent; it also, helps in inducing the red-light production of *CCA 1/LHY*. It mainly helps in synchronizing the signaling of light from PHY to outputs of the clock. It also has a role in controlling gene expression and light-dependent development processes.	Más et al., 2003

## Regulation of Photosynthesis by Circadian Rhythm

The continual pattern of the circadian rhythm in many organisms, mainly in plants, leads to the opening of stomata during daytime and the closing of stomata during the nighttime (Webb, 2003). The circadian rhythm plays an essential role in the interaction between photosynthesis activities and the diurnal variation based upon the availability of light (Hennessey and Field, 1991). The minute opening in the leaves is said to be stomata; thus, the opening and closing of stomata that concern the balanced amount of carbon dioxide taken inside along with the proportion of water and oxygen are released out. Photosynthesis itself is a salient circadian rhythm in the plant, which is composed of various molecular and physiological processes, such as stomatal opening, chlorophyll contents, chlorophyll fluorescence, and net carbon assimilation rate (Rascher et al., 2001; Pan et al., 2015).

The opening of stomata is an essential process for net carbon assimilation. It is also essential for the carbon dioxide diffusion into mesophyll cells from the atmosphere, based on the cost of water. Naturally, photosynthesis production gets affected by the action of circadian responsiveness or closing of stomata (Youngsung et al., 2017). Furthermore, the rhythm produced in stomatal conductance could affect the carbon assimilation rate by restricting the flow of carbon dioxide into the leaves. The process of photosynthesis influences plant metabolism; thus, it synchronizes the Photosystem II activity or stomatal opening and movement of the chloroplast. From the previous study on *A. thaliana* (Dodd et al., 2015), the overexpression of *CCA1* in the plant could lead to the reduction of net carbon assimilation, which may also lead to the reduction of net carbon assimilation maximal plant fitness.

There is a bidirectional relationship between the biological clock and photosynthesis because photosynthesis gets affected by circadian regulation. In addition, the circadian clock has a core structure due to which it gets affected by photosynthesis (Haydon et al., 2013). The regulation of this oscillator affects the diurnal fluctuation in the stomatal conductance and photosynthesis process and various processes, such as respiration and growth. Even the behavior of stomata gets affected by circadian regulation (de Dios et al., 2020). The fraction of the diurnal pattern of variation in the stomatal conductance, which is allocated to the clock, is higher in amount during the changes in the daytime than in the changes allocated to the clock by photosynthesis. Thus, this indicates that the stomatal conductance has a vigorous circadian regulation than photosynthesis (de Dios et al., 2018).

Several studies on *A. thaliana* found that the endogenous rhythm controls photosynthesis and physiology, and the plant attains a peak of fitness (Green et al., 2002; Dodd et al., 2005; Müller et al., 2014). The regulation of the circadian oscillator raises the plant productivity, viability of seed, and plant survival. From de Dios et al. (2018), it is known that, in the Calvin cycle, the rate of photosynthesis depends upon the stomatal conductance and the mesophyll conductance and the biochemical process. The adjustment made in the accumulation of carbon dioxide present within the intercellular spaces by the stomatal conductance helps in modulating the process of photosynthesis. It is also known that there is a negative interaction or lack of interaction between photosynthesis and stomatal conductance. This suggests that the circadian regulation in stomatal conductance is implausible to guide the regulation of photosynthesis by circadian oscillation (Males and Howard, 2017). There is a primary role for endogenous rhythm in mesophyll conductance in balancing the accumulation of carbon dioxide in the chloroplast.

An endogenous rhythm in the framework of the light-harvesting compounds is an essential feature for the circadian rhythm in photosynthesis. The chlorophyll synthesis rate is controlled by the circadian rhythm and diurnal variations in chlorophyll a/b, which is also connected with an endogenous rhythm in the process of photosynthesis. The gene light-harvesting complex A/B protein (LHCB) activation occurs when the CCA1 and LHY moderate; it also directly gets binding with the promoters. The circadian regulation synchronizes the gene transcription linked with the Calvin cycle. Photosynthesis interacts with the *PRR7* gene to manage the maintenance and entrainment of vigorous circadian oscillators (Haydon et al., 2013). In addition to this, the expression level of LHY/CCA1 and PRR7/PRR9 has the capability to induce the performance of plant growth and development (Müller et al., 2014).

In the C3 photosynthesis pathway or photosynthetic carbon reduction (PCR) cycle (Furbank and Taylor, 1995), plants consist of a single chloroplast that conducts many reactions; mainly, it helps in the conversion of light energy into chemical energy. Ribulose-1, 5-bisphosphate carboxylase/oxygenase (RuBisCO) is the protein used to activate the initial fixation of carbon called ribulose-1, 5-bisphosphate (RuBP), which is a five-carbon sugar-phosphate, where carbon dioxide is getting converted as two molecules in a three-carbon compound called 3-phosphoglyceric acid (PGA) or 3-carbon acid (Locke et al., 2018). It is also found that the RuBisCO protein dispersed inside the chloroplast changes according to the rhythmic duration; additionally, this dispersal of RuBisCO protein interacts with the rhythm of carbon dioxide fixation (Nassoury et al., 2001).

During the daytime, nicotinamide adenine dinucleotide phosphate (NADPH) and adenosine triphosphate (ATP) are produced with the help of a light reaction to charge the assimilation of carbon. The carbon present, in addition, is deposited as starch inside the chloroplast, which ends up as the diurnal variations of starch concentration and reduction (Locke et al., 2018). Photorespiration is the process where the oxygen fixation gets activated, and then it directly takes place in the carbon dioxide fixation. The coherence of C3 photosynthesis in the air can be estimated based on the conflict between carbon dioxide and oxygen and the cost of energyrelated by reclaiming phosphoglycolate. In C4 plants, photosynthesis occurs where NADP-malic enzyme (ME) helps transfer the malate from mesophyll to bundle sheath chloroplast.

Jones (2017) described that crassulacean acid metabolism (CAM) photosynthetic pathway allows enhanced productivity of water usage by isolating the carbon dioxide fixation for the opening of stomata during the daytime, and it closes the stomata and controls the water loss. Thus, these CAM plants open their stomata and fix the carbon dioxide with the aid of phosphoenolpyruvate carboxylase (PPC) and malate dehydrogenase activity during nighttime (Boxall et al., 2020). The activity of PPC has synchronized the mixture of PPC with circadian-regulated expression along with the PPC phosphorylation, which further ends up changing the activity of carboxylase. The circadian oscillator-related PPC activity initiates malate, then deposited into vacuoles as the malic acid (Hartwell et al., 2016). During the night, malate comes out of vacuoles. It enters the cytosol, where the decarboxylation takes place with the help of an enzyme Phosphopyruvate Carboxykinase (PCK) or by NAD-malic enzyme (NAD-ME) on CAM variants. This could further lead to an increase in the level of carbon dioxide in leaves, which influences the stomatal closure authorizing RuBisCO to return the carbon dioxide into the Calvin Benson cycle in the presence of light.

## Regulation of Carbon Metabolism by Circadian Oscillations

The circadian oscillation has been intimated for modulating many enzymes included in plant carbon (primary) metabolism (Harmer et al., 2000). The photosynthate inflation notifies the advancement of this biological system. The providence of carbon plays an essential role in plants while scrutinizing the different methods for improving crop productivity. Many different enzymes are involved in isolation during carbon metabolisms, such as chlorophyll a/b binding protein (CAB), RuBisCO (RBCS), phosphoenopyruvate carboxykinase (PEPC-K), phosphoenolpyruvate carboxylase (PEPC), ME, pyruvate kinase (PPDK), sucrose phosphate synthase (SPS), pyruvate kinase (PK), sucrose-non-fermentation1-related protein kinase1 (SnRK1), and the DNA-binding with one finger (Dof) co-expression; these were the genes looked over during the diurnal conditions (Kanwal et al., 2014).

Generally, in carbon metabolism, the efficacy of light is considered a driving force, and an entraining signal of the Calvin-Benson-Bassham (CBB) cycle or C3 cycle tries to isolate their parts. These spotted genes have recognized an endogenous rhythm present in the genotypes and the various patterns during the high range of expression. At the time of dawn, the compulsion of photosynthetic carbon dioxide directs the sucrose synthesis and the accumulation of starch; thus, it assists for the pursued production of starch during the time of dusk (Graf and Smith, 2011; Stitt and Zeeman, 2012; Pokhilko and Ebenhoh, 2015; Kim et al., 2017). At dusk, the starch stockpiled throughout the day before night will be deteriorated and devoured. Starch yield disruption occurs due to an unpredicted rapid arrival of dusk, which leads to the early consumption of starch and malnourishment of carbon. This malnourishment of carbon can end up in the swift alternate in gene expression; metabolism can lead to the minute difference in the rate of growth, which could further result in a higher difference in biomass production within a few weeks. It is feasible to directly or indirectly manage these mechanisms of endogenous clock genes (Lu et al., 2005; Graf et al., 2010; Kotting et al., 2010). From previous (Sulpice et al., 2014) studies, it is known that the clock incidentally regulates the synthesis of starch by the modulation of starch degradation.

Mostly, in plants, the amount of carbon secured along with starch is higher in level when compared to wild type depending upon the decreasing range of circadian rhythm period (K€olling et al., 2015; Jones, 2017). They reveal the transcriptional alternation, which is an indication for the starvation of carbon compounds before night. The plants were usually grown in a 12-h light and 12-h dark period for 3 continual weeks. It is known from Graf et al. (2010), when the plants are further relocated into dark conditions only after the 8 h of light exhibited a high range of starch degradation, and also there was no indication of carbon starvation found throughout the next 16 h of dark condition. In well-sustained plants, when the plant growth is strictly restricted by the carbon supply then the growth is managed either by CCA1 or LHY (Yazdanbakhsh et al., 2011; Müller et al., 2014).

The oilseed and vegetable varieties have various morphologies and different harvestable sections. This oilseed type of crop has a minimal period of circadian rhythm and maximal range of net carbon assimilation when compared to the vegetable varieties (Yarkhunova et al., 2016). From Smith and Stitt (2007), it is known that the rate of photosynthesis becomes limited under the condition of a shorter duration of photoperiods (or low irradiance). In such a case, it could lead to a limited source phenotype, which is the condition of insufficient or lack of carbon and minimal plant growth. Whereas, whenever more carbon is obtained from the atmosphere for the better growth of plants, having higher irradiance or long timing of photoperiod and the plants are of limited sink phenotype.

The clock maintains a certain level of carbon supply rate with the help of clock component CCA1/LHY, which could be further stored and used until the next day. During the nighttime, the instability of starch can be managed by the clock component CCA1/LHY in *A. thaliana*. This regulation plays a major role in avoiding either the lack or breakdown of sucrose and decreased growth level during nighttime (Müller et al., 2014). The rhythmic clock component TOC1 provides a specific idea for the biological system in the maintenance of cellular mechanisms (Cervela-Cardona et al., 2021).

## Regulation of Sugar Metabolism by the Circadian Clock

Due to the fluctuation in the photoperiod, the rise in sugars changes, that is, the increase or decrease of sugars depends upon the presence of light and photosynthesis rate. Thus, it indicates sugar does not increase in the early morning or in late evening; also clock loses the sensitivity at the end of the light period, as it is not phase advanced (Haydon et al., 2013). The early rise of sugar takes place on sunny days, which helps the circadian clock in showing the appropriate phase for driving processes that are linked with sugar metabolism. Sugar accumulation in the morning helps in the activation of the photosynthesis process on all days of the cycle.

Due to sugars, the activation of the CCA1 promoter increases; also simultaneously, it also suppresses the PRR7 (Haydon et al., 2013; Dodd et al., 2015). The regulation of sugar with the circadian clock has the capability to develop the action of the oscillator for dealing with the clock timing; this may also form a mechanism where biological processes, which are considered as the outputs of biological oscillators, are getting affected by sugars. These all are because sugars can manage the outputs of the circadian clock directly, even without performing any role in the core region of the clock. The circadian clock may get affected by sugars in long-term response pathway, which strengthens the rhythmicity in leaves of *A. thaliana* during the nighttime, after which there will be a need for GI (Dalchau et al., 2010).

With the help of PHY and CRY photoreceptors, the entrainment cues, including signals of light, are obtained. It also comprises the regulation of FLOWERING LOCUS T (FT) through the sugar signaling of Trehalose-6-Phosphate (T6P); even though the photoperiod synchronization of FT is not totally dependent upon this T6P (Dodd et al., 2015). In the late daytime, during the process of photosynthesis, sugars are synthesized and these sugars are then conserved and further exploited in the process of respiration during the nighttime. On the basis of different carbohydrate transports along with the various stages of cycle rhythmicity, it develops the diel oscillation of sugars (Haydon et al., 2011).

## Regulation of Flowering and Senescence in Plants by Circadian Rhythm

The development of flowers is an important stage in plant development. The formation of flower organs occurs when the gene expressions are accurately controlled during the flower development stage (Schaffer et al., 1998). This process is activated by the circadian rhythm (Schaffer et al., 1998). The flowering genotype depends upon the merged reactions of external and internal features, such as photoperiod and temperature (Min, 2005). The clock recognizes the length of photoperiod and regulates the phloem companion cells FT and CONSTANS (*CO*); where CO is a cue regulator for flowering when there is a conflict between the length of photoperiod and the circadian rhythm based on external coincidence theory (Sawa et al., 2007). The leaf senescence regulators *ORESARA 1 (ORE 1)* are controlled by endogenous rhythm and activated by various other components, such as *CCA1*, *PRR9*, *ELF3*, *ELF4*, and *LUX*.

The *ORE 1* gene is present downstream of *ETHYLENE INSENSITIVE 2 (EIN 2*), which activates the ethylene signaling pathway. This *ORE 1* gene encodes with protein-specific transcriptional factor (TF) NAM, ATAF, and CUC (NAC), which then positively activates the senescence in leaves and moderates various pathways of leaf senescence by activating *SENESCENCE ASSOCIATED GENES* (*SAGs*) expression, such as *SAG 29* and *BIFUNCTIONAL NUCLEASE 1* (*BFN 1*) (Matallana-Ramirez et al., 2013). The *ORE 1* expression increases during cell aging, leading to age-induced cell apoptosis. The microRNA164 (miR164) negatively activates the expression of *ORE 1*. The trifurcate feed-forward loop is formed with the combination of *EIN 2*, miR164, and *ORE 1*, which changes the senescence of the leaf (Kim et al., 2009). During leaf aging, the circadian rhythm gets shortened, which is found in the model organism *Arabidopsis* (Kim et al., 2016). From the previous study, it is known that the duration of flowering in *Arabidopsis* takes long days (LDs) and also its mechanism is well explained (Corbesier and Coupland, 2005).

**FIGURE 2:**
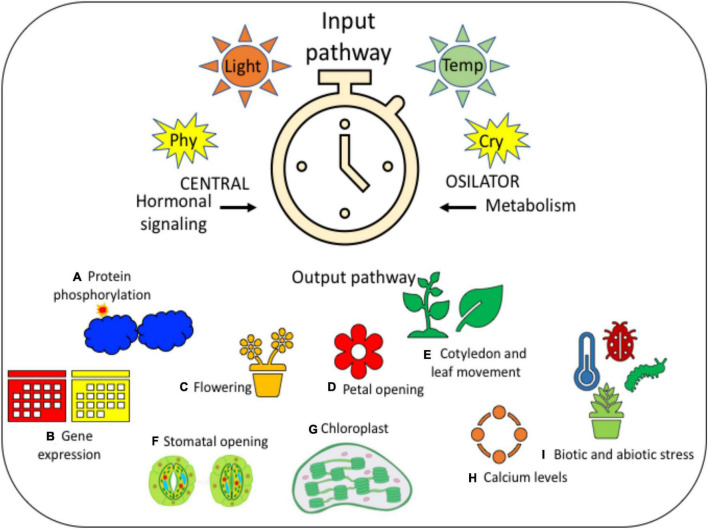
It is a diagrammatic representation of the circadian rhythm for providing a clear idea on the same. It has three different stages, such as (i) input pathway that contains light and temperature, which is then followed by (ii) central oscillator, in the presence of photoreceptors, such as PHY and CRY; further leading to the (iii) output pathway, which consists of many different physiological and developmental processes, such as **(A)** protein phosphorylation, **(B)** gene expression, **(C)** flowering, **(D)** petal opening, **(E)** cotyledon and leaf movement, **(F)** stomatal opening, **(G)** chloroplast, **(H)** calcium levels, and **(I)** biotic and abiotic stress. All these processes indicate the step-by-step variations that take place in every plant during the changes in photoperiodic rhythm.

In *A. thaliana*, the GI, which is the component of the clock, activates the photoperiodic flowering. This GI binds along with the CO promoter to activate the expression of *CO* (Figure 2). GI controls leaf senescence as the time of flowering, where it depends on the location. GI often acts as the mediator to make the interaction between leaf senescence and flowering and develops the productivity and fitness of plants. The *ELF 4* links directly with the GI and suppresses its interaction with the ORE 1 promoter (Kim et al., 2020). The result of the regulatory network controls the transcription of CO, and CO mRNA gets collected after the night in a short-time duration. Then CO stops to assemble in the dusk due to degradation of protein by the combination of CONSTITUTIVE PHOTOMORPHOGENESIS 1 (COP 1) and SUPPRESSOR OF PHYA-105 1 (SPA 1) (Laubinger et al., 2006; Jang et al., 2008). The CRYPTOCHROME 2 (CRY 2), which is photoactivated, combines with COP 1 and SPA 1 to make a complex compound to inhibit the degradation of CO (Zuo et al., 2011).

Furthermore, by forming a blue light-dependent complex with FKF1 (FLAVIN-BINDING, KELCH REPEAT, F-BOX 1), GI regulates the expression of floral activators CO and FT (Imaizumi et al., 2003, 2005; Sawa et al., 2007; Sawa and Kay, 2011). CYCLING DOF FACTOR 1 (CDF1), a protein that represses CO and FT transcription, is degraded by the GI-FKF1 complex (Imaizumi et al., 2005; Fornara et al., 2009; Song et al., 2012). CO protein levels, which are stabilized by FKF1 at the end of the long photoperiod, control the activation of FT expression in LDs (Suárez-López et al., 2001; Song et al., 2012). Furthermore, light regulates CO protein, so CO levels are low in red light and high in blue light. While blue and far-red light help to keep CO stable (Valverde et al., 2004). This control increases the floral signal by reinforcing the accumulation of CO protein levels in the evening of LDs. These molecular interactions theoretically result in a double external coincidence mechanism that involves multiple clock outputs, but the combined effects of this rhythmic mechanism have not been quantitatively tested or incorporated into previous mathematical models (Song et al., 2012).

Similarly, the senescence in plants requires a functional endogenous system; also, many different senescence-related genes encode the TFs in which NAC and WRKY show a clear circadian rhythm pattern (Kim and Hong, 2019). The clock rhythm has combined the function of both flowering and leaf senescence, due to which the early leaf senescence takes place because of the ELF period as per the causes of embryonal carcinoma (EC) mutants (Kim et al., 2018). The analysis of transcriptional profiling exhibits the senescence regulatory genes; not only the *ORE 1*, *NAP*, *WRKY 53*, and *WRKY 70* but also the phytohormone jasmonate (JA) responsive and signaling genes are getting activated at the stage of EC-mediated senescence of leaf (Zhang et al., 2018). The salicylic acid (SA) pathway has a connection with the PHY-associated leaf senescence mechanism. The regulation of senescence on the basis of circadian oscillator and light has been explained in a detailed manner in a recent study (Lee et al., 2021).

## Defense Response in Plants by Circadian Rhythms

The circadian rhythm can also be expressed with the help of the plant defense system, also in the absence of the pathogen. The glycine-rich RNA binding protein is a pathogen responsive gene, and it was already seen in several crops that exhibit the regulation of the clock (Lu et al., 2017). Defense response plays a significant role in plants for the normal functioning of cells against the infection caused by the pathogen (Spoel and Dong, 2008). In the case of a pathogen attack, the cuticle supply, which is a physical plant barrier, is considered the first line of plant defense. This form of defense system blocks the pest and pathogen invasion. Whenever a swap takes place in the cuticle, it is immediately realized by the plant and instantly, it begins the defense response during the time of pathogen attack (Malik et al., 2020). The instigated interaction, which occurs between pattern recognition receptors and pathogen-associated molecular patterns (PAMPs), activates a considerable amount of plant defense systems, such as stomatal closure, to keep away from the invasion of the pathogen (Jones and Dangl, 2006; Miller et al., 2017; Butt et al., 2020).

Phytohormones, such as JA and SA, play an essential role in defense responses (Roden and Ingle, 2009; Tamaoki et al., 2013). Depending upon the mode of pathogen attack, the phytohormones are synthesized by the plants (Loake and Grant, 2007). If the pathogen moves inside the plant cells, the polymorphic Nucleotide Binding and Leucine-Rich Repeat (NB-LRR) proteins available within the host cell interconnect with specifically effecting molecules of the pathogen (Jones and Dangl, 2006; Miller et al., 2017). The growth by the addition of SA influences the strengthening of the cell wall, phenolic accumulation, and production. It also triggers resistance (R) and other different defense response genes (activated of clock gene *LUX*). The PRRs recognize the PAMPs that stop the formation of pathogen colonies, which end up in the PAMP triggered immunity (PTI). This PTI restricts the released action of the pathogen inside the host cell, and also it switches on the defense system, which functions in the way of non-host-specific R. The four stages of plant immunity have become a well-known process from a recent study (Butt et al., 2020).

The prompt in reactive oxygen species (ROS) production is considered the initial defense response state in plants. The interaction between the clock system and redox state in the cellular system confirms the stability between the immunity of plants and their proper growth (Brody, 2019; Zhang J. et al., 2019). The *LUX* gets bonded with EDS 1 and JASMONATE ZIM DOMAIN (JAZ) 5 promoters, which could affect the signaling of JA and SA (Zhang C. et al., 2019). The formation of co-receptor complex takes place with the association of transcriptional repressor proteins, such as JASMONOYL-ISOLEUCINE (JA-Ile), JAZ, and CORONATINE-INSENSITIVE1 (COI1), which is an F-Box protein in the existence of vital appearance of JA (Nitschke et al., 2016). This leads to the JAZ repressor degradation due to the ubiquitin-proteasome system (Thines et al., 2007), and then it rescues the TFs, such as MYC 2. Thus, the transcription for response genes of JA is enabled, such as JAZ genes, also MYC 2, and all the biosynthesis genes of JA, i.e., *LIPOXYGENASE* (*LOX*) and *OXO PHYTODIENOATE REDUCTASE 3* (*OPR3*) (Chung et al., 2008; Wasternack and Hause, 2013).

The clock entrainment with the light is conciliated by CRY and PHY; they are translocated to the nucleus concerning the light where the gene expression is activated. Several defense genes are moderated by the function of PHY and the circadian clock (Roden and Ingle, 2009). Plants having the deficiency of PHY A/B show the complete reduction in the expression of PRR; thus, this stipulates the importance of the defense signaling pathway (Genoud et al., 2002; Butt et al., 2020). From Griebel and Zeier (2008), it is known that the plant defense has an increased function during the time of dawn rather than dusk; also, they have used the pressure inoculum in the leaves of the organism *Pseudomonas syringae* (*P. syringae*) along with the diversion of defense response in stomata. The rhythmicity of the clock blocks the susceptibility of the plants toward many different pathogens and insects, where the plant susceptibility will have the most little part at the time of interaction of endogenous clock with the defense hormones, such as JA and SA (Goodspeed et al., 2012; Korneli et al., 2014; Ingle et al., 2015).

## Conclusion

The plant circadian rhythm is cell-independent and self-assisting under the seasonal and diurnal input sources from the environmental cue for encountering the stimulant-navigated response. The endogenous clock components will be synchronized at either transcriptional or post-transcriptional extent. The role and the function of *CCA1* and *LHY* (the two main clock components in light signaling) in circadian timing and control of the flowering plant could be explained with the separation and depiction of clock genes. It has been found that after dawn, the genes help much in controlling the chlorophyll; it was also known that these clock components help maintain the fitness of plants with improved immunity for the best performance.

In the present review, we have tried to explain the different biological or developmental processes of plants through the circadian clock, which will also help the researchers understand how the genes present in the circadian system, are expressed, and how they are functioning. The circadian rhythm has been analyzed with the model organism *A. thaliana* and in other plants, such as *Nicotiana benthamiana* (*N. benthamiana*), *Chrysanthemum*, and *Petunia* plant. Yet, there are many vegetable crops or flowering plants, which could be readily available. In addition, it can be used to analyze circadian biology to provide a clear vision about the same in a very detailed manner. Both *in vitro* and *in vivo* studies for the analysis of this photoperiodic rhythmicity along with the abiotic stress conditions using molecular work can be performed as a brief future study. The study can be accomplished to increase the growth and yield of edible crops that are highly tolerant toward the abiotic stresses, for example, salinity, heat, and cold stress conditions, based on the functioning of their circadian rhythm.

## Author Contributions

All authors listed have made a substantial, direct, and intellectual contribution to the work, and approved it for publication.

## Conflict of Interest

The authors declare that the research was conducted in the absence of any commercial or financial relationships that could be construed as a potential conflict of interest.

## Publisher’s Note

All claims expressed in this article are solely those of the authors and do not necessarily represent those of their affiliated organizations, or those of the publisher, the editors and the reviewers. Any product that may be evaluated in this article, or claim that may be made by its manufacturer, is not guaranteed or endorsed by the publisher.

## References

[B1] Aguilar-Arnal L. Sassone-Corsi P. (2015). Chromatin landscape and circadian dynamics: Spatial and temporal organization of clock transcription. *Proc. Nat. Acad. Sci. U S A.* 112 6863–6870. 10.1073/pnas.1411264111 25378702 PMC4460512

[B2] Belbin F. E. Hall G. J. Jackson A. B. Schanschieff F. E. Archibald G. Formstone C. (2019). Plant circadian rhythms regulate the effectiveness of a glyphosate-based herbicide. *Nature Commun.* 10 1–1. 10.1038/s41467-019-11709-5 31420556 PMC6697731

[B3] Boxall S. F. Kadu N. Dever L. V. Kneřová J. Waller J. L. Gould P. J. D. (2020). Kalanchoë PPC1 is essential for crassulacean acid metabolism and the regulation of core circadian clock and guard cell signaling genes. *Plant Cell* 32 1136–1160. 10.1105/tpc.19.00481 32051209 PMC7145507

[B4] Brody S. (2019). Circadian Rhythms in Fungi: Structure/Function/Evolution of Some Clock Components. *J. Biol. Rhythm* 34 364–379. 10.1177/0748730419852832 31216909

[B5] Butt G. R. Qayyum Z. A. Jones M. A. (2020). Plant Defence Mechanisms Are Modulated by the Circadian System. *Biology* 9:454. 10.3390/biology9120454 33317013 PMC7763185

[B6] Cervela-Cardona L. Alary B. Mas P. (2021). The *Arabidopsis* circadian clock and metabolic energy: A question of time. *Front. Plant Sci.* 12:804468. 10.3389/fpls.2021.804468 34956299 PMC8695440

[B7] Chen Y. Y. Wang Y. Shin L. J. Wu J. F. Shanmugam V. Tsednee M. (2013). Iron is involved in the maintenance of circadian period length in *Arabidopsis*. *Plant Physiol.* 161 1409–1420. 10.1104/pp.112.212068 23307650 PMC3585605

[B8] Chung H. S. Koo A. J. Gao X. Jayanty S. Thines B. Jones A. D. (2008). Regulation and function of *Arabidopsis* JASMONATE ZIM-domain genes in response to wounding and herbivory. *Plant Physiol.* 146 952–964. 10.1104/pp.107.115691 18223147 PMC2259048

[B9] Corbesier L. Coupland G. (2005). Photoperiodic flowering of Arabidopsis: integrating genetic and physiological approaches to characterization of the floral stimulus. *Plant Cell Environ.* 28 54–66. 10.1111/j.1365-3040.2005.01283.x

[B10] Dalchau N. Baek S. J. Briggs H. M. Robertson F. C. Dodd A. N. Gardner M. J. (2011). The circadian oscillator gene GIGANTEA mediates a long-term response of the *Arabidopsis thaliana* circadian clock to sucrose. *Proc. Nat. Acad. Sci. U S A.* 108 5104–5109. 10.1073/pnas.1015452108 21383174 PMC3064355

[B11] Dalchau N. Hubbard K. E. Robertson F. C. Hotta C. T. Briggs H. M. Stan G. B. (2010). Correct biological timing in Arabidopsis requires multiple light-signaling pathways. *Proc. Nat. Acad. Sci. U S A.* 107 13171–13176. 10.1073/pnas.1001429107 20615944 PMC2919914

[B12] de Dios V. R. Anderegg W. R. L. Li X. Tissue D. T. Bahn M. Damien L. (2020). Circadian regulation does not optimize stomatal behaviour. *Plants* 9:1091. 10.3390/plants9091091 32854373 PMC7570086

[B13] de Dios V.R. Anderegg W. R. L. Li X. Tissue D. T. (2018). Circadian regulation of photosynthesis and transpiration from genes to ecosystems. *Environ. Exp. Bot.* 152 37–48. 10.1016/j.envexpbot.2017.09.010

[B14] Dodd A. N. Belbin F. E. Frank A. Webb A. A. R. (2015). Interactions between circadian clocks and photosynthesis for the temporal and spatial coordination of metabolism. *Front. Plant Sci.* 6:245. 10.3389/fpls.2015.00245 25914715 PMC4391236

[B15] Dodd A. N. Salathia N. Hall A. Ke’vei E. To’th R. Nagy F. (2005). Plant circadian clocks increase photosynthesis, growth, survival, and competitive advantage. *Science* 309 630–633. 10.1126/science.1115581 16040710

[B16] Farré E. M. Harmer S. L. Harmon F. G. Yanovsky M. J. Kay S. A. (2005). Overlapping and distinct roles of PRR7 and PRR9 in the *Arabidopsis* circadian clock. *Curr. Biol.* 15 47–54. 10.1016/j.cub.2004.12.067 15649364

[B17] Feke A. Vanderwall M. Liu W. Gendron J. M. (2021). Functional domain studies uncover novel roles for the ZTL Kelch repeat domain in clock function. *PLoS One* 16:e0235938. 10.1371/journal.pone.0235938 33730063 PMC7968664

[B18] Fornara F. Panigrahi K. C. S. Gissot L. Sauerbrunn N. Rühl M. Jarillo J. A. (2009). Arabidopsis DOF transcription factors act redundantly to reduce CONSTANS expression and are essential for a photoperiodic flowering response. *Dev. Cell* 17 75–86. 10.1016/j.devcel.2009.06.015 19619493

[B19] Franklin K. A. (2020). PRR proteins of the circadian clock call time on shade avoidance. *Proc. Nat. Acad. Sci. U S A.* 117 5095–5096. 10.1073/pnas.2000716117 32060121 PMC7071862

[B20] Fukuda N. Suenaga T. Miura E. Tsukamoto A. (2020). The Expression of ELF4-Like Genes Is Influenced by Light Quality in *Petunia*. *Agronomy* 10:1800. 10.3390/agronomy10111800

[B21] Furbank R. T. Taylor W. C. (1995). Regulation of photosynthesis in C3 and C4 plants: a molecular approach. *Plant Cell* 7:797. 10.1105/tpc.7.7.797 12242386 PMC160868

[B22] Genoud T. Buchala A. J. Chua N. H. Métraux J. P. (2002). Phytochrome signalling modulates the SA-perceptive pathway in *Arabidopsis*. *Plant J.* 31 87–95. 10.1046/j.1365-313x.2002.01338.x 12100485

[B23] Goodspeed D. Chehab E. W. Min-Venditti A. Braam J. Covington M. F. (2012). *Arabidopsis* synchronizes jasmonate-mediated defense with insect circadian behavior. *Proc. Nat. Acad. Sci. U S A.* 109 4674–4677. 10.1073/pnas.1116368109 22331878 PMC3311395

[B24] Graf A. Smith A. M. (2011). Starch and the clock: the dark side of plant productivity. *Trends Plant Sci.* 16 169–175. 10.1016/j.tplants.2010.12.003 21216654

[B25] Graf A. Schlereth A. Stitt M. Smith A. M. (2010). Circadian control of carbohydrate availability for growth in *Arabidopsis* plants at night. *Proc. Nat. Acad. Sci. U S A.* 107 9458–9463. 10.1073/pnas.0914299107 20439704 PMC2889127

[B26] Green R. M. Elaine M. T. (2002). The role of CCA1 and LHY in the plant circadian clock. *Dev. Cell* 2 516–518. 10.1016/s1534-5807(02)00184-3 12015957

[B27] Green R. M. Tingay S. Wang Z. Y. Tobin E. M. (2002). Circadian rhythms confer a higher level of fitness to *Arabidopsis* plants. *Plant Physiol.* 129 576–584. 10.1104/pp.004374 12068102 PMC161679

[B28] Griebel T. Zeier J. (2008). Light regulation and daytime dependency of inducible plant defenses in Arabidopsis: phytochrome signaling controls systemic acquired resistance rather than local defense. *Plant Physiol.* 147 790–801. 10.1104/pp.108.119503 18434604 PMC2409012

[B29] Harmer S. L. (2009). The circadian system in higher plants. *Annu. Rev. Plant Biol.* 60 357–377. 10.1146/annurev.arplant.043008.092054 19575587

[B30] Harmer S. L. Hogenesch J. B. Straume M. Chang H. S. Han B. Zhu T. (2000). Orchestrated transcription of key pathways in *Arabidopsis* by the circadian clock. *Science* 290 2110–2113. 10.1126/science.290.5499.2110 11118138

[B31] Hartwell J. Dever L. V. Boxall S. F. (2016). Emerging model systems for functional genomics analysis of Crassulacean acid metabolism. *Curr. Opin. Plant Biol.* 31 100–108. 10.1016/j.pbi.2016.03.019 27082281

[B32] Haydon M. J. Bell L. J. Webb A. A. (2011). Interactions between plant circadian clocks and solute transport. *J. Exp. Bot.* 62 2333–2348. 10.1093/jxb/err040 21378117

[B33] Haydon M. J. Mielczarek O. Robertson F. C. Hubbard K. E. Webb A. A. R. (2013). Photosynthetic entrainment of the *Arabidopsis thaliana* circadian clock. *Nature* 502 689–692. 10.1038/nature12603 24153186 PMC3827739

[B34] Hennessey T. L. Field C. B. (1991). Circadian rhythms in photosynthesis: oscillations in carbon assimilation and stomatal conductance under constant conditions. *Plant Physiol.* 96 831–836. 10.1104/pp.96.3.831 16668261 PMC1080851

[B35] Hicks K. A. Tina M. A. Wagner D. R. (2001). EARLY FLOWERING3 encodes a novel protein that regulates circadian clock function and flowering in *Arabidopsis*. *Plant Cell* 13 1281–1292. 10.1105/tpc.13.6.1281 11402160 PMC135582

[B36] Huq E. Tepperman J. M. Quail P. H. (2000). GIGANTEA is a nuclear protein involved in phytochrome signaling in *Arabidopsis*. *Proc. Nat. Acad. Sci. U S A.* 97 9789–9794. 10.1073/pnas.170283997 10920210 PMC16943

[B37] Imaizumi T. Schultz T. F. Harmon F. G. Ho L. A. Kay S. A. (2005). FKF1 F-box protein mediates cyclic degradation of a repressor of CONSTANS in Arabidopsis. *Science* 309 293–297. 10.1126/science.1110586 16002617

[B38] Imaizumi T. Tran H. G. Swartz T. E. Briggs W. R. Kay S. A. (2003). FKF1 is essential for photoperiodic-specific light signalling in Arabidopsis. *Nature* 426 302–306. 10.1038/nature02090 14628054

[B39] Ingle R. A. Stoker C. Stone W. Adams N. Smith R. Grant M. (2015). Jasmonate signalling drives time-of-day differences in susceptibility of *Arabidopsis* to the fungal pathogen Botrytis cinerea. *Plant J.* 84 937–948. 10.1111/tpj.13050 26466558 PMC4982060

[B40] Jang S. Marchal V. Panigrahi K. C. Wenkel S. Soppe W. Deng X. W. (2008). *Arabidopsis* COP1 shapes the temporal pattern of CO accumulation conferring a photoperiodic flowering response. *EMBO J.* 27 1277–1288. 10.1038/emboj.2008.68 18388858 PMC2291449

[B41] Jones J. Dangl J. (2006). The plant immune system. *Nature* 444 323–329. 17108957 10.1038/nature05286

[B42] Jones M. A. (2017). Interplay of circadian rhythms and light in the regulation of photosynthesis-derived metabolism. *Progress Bot.* 79 147–171. 10.1007/124_2017_2 33311142

[B43] K€olling K. Thalmann M. Müller A. Jenny C. Zeeman S. C. (2015). Carbon partitioning in *Arabidopsis thaliana* is a dynamic process controlled by the plants metabolic status and its circadian clock. *Plant Cell Environ.* 38 1965–1979. 10.1111/pce.12512 25651812 PMC4671261

[B44] Kanwal P. Gupta S. Arora S. Kumar A. (2014). Identification of genes involved in carbon metabolism from *Eleusine coracana* (L.) for understanding their light-mediated entrainment and regulation. *Plant Cell Rep.* 33 1403–1411. 10.1007/s00299-014-1625-4 24825394

[B45] Inoue K. Araki T. Endo M. (2017). Integration of input signals into the gene network in the plant circadian clock. *Plant Cell Physiol.* 58 977–982. 10.1093/pcp/pcx066 27095830

[B46] Inoue K. Araki T. Endo M. (2018). Circadian clock during plant development. *J. Plant Res.* 131 59–66. 10.1007/s10265-017-0991-8 29134443 PMC5897470

[B47] Kiełbowicz-Matuk A. Rey P. Rorat T. (2014). Interplay between circadian rhythm, time of the day and osmotic stress constraints in the regulation of the expression of a Solanum Double B-box gene. *Ann. Bot.* 113 831–842. 10.1093/aob/mct303 24562097 PMC3962237

[B48] Kim C. Kim S. J. Jeong J. Park E. Oh E. Park Y. I. (2020). High ambient temperature accelerates leaf senescence *via* PHYTOCHROME-INTERACTING FACTOR 4 and 5 in *Arabidopsis*. *Mol. Cells* 43:645. 10.14348/molcells.2020.0117 32732458 PMC7398796

[B49] Kim H. Hong S. (2019). Role of the circadian clock in fine-tuning the process of leaf senescence in plants. *Translat. Med. Aging* 3 26–30. 10.1016/j.tma.2018.12.001

[B50] Kim H. Kim H. J. Vu Q. T. Jung S. McClung C. R. Hong S. (2018). Circadian control of ORE1 by PRR9 positively regulates leaf senescence in *Arabidopsis*. *Proc. Nat. Acad. Sci. U S A.* 115 8448–8453. 10.1073/pnas.1722407115 30065116 PMC6099856

[B51] Kim H. Kim Y. Yeom M. Lim J. Nam H. G. (2016). Age-associated circadian period changes in *Arabidopsis* leaves. *J. Exp. Bot.* 67 2665–2673. 10.1093/jxb/erw097 27012281 PMC4861015

[B52] Kim J. A. Kim H. S. Choi H. S. Jang J. Y. Jeong M. J. Lee S. I. (2017). The importance of the circadian clock in regulating plant metabolism. *Int. J. Mol. Sci.* 18:2680. 10.3390/ijms18122680 29232921 PMC5751282

[B53] Kim J. H. Woo H. R. Kim J. Lim P. O. Lee I. C. Choi S. H. (2009). Trifurcate feed-forward regulation of age-dependent cell death involving miR164 in *Arabidopsis*. *Science* 323:1053e1057. 10.1126/science.1166386 19229035

[B54] Korneli C. Danisman S. Staiger D. (2014). Differential control of pre-invasive and post-invasive antibacterial defense by the *Arabidopsis* circadian clock. *Plant Cell Physiol.* 55 1613–1622. 10.1093/pcp/pcu092 24974385

[B55] Kotting O. Kossmann J. Zeeman S. C. Lloyd J. R. (2010). Regulation of starch metabolism: The age of enlightenment? *Curr. Opin. Plant Biol.* 13 321–329. 10.1016/j.pbi.2010.01.003 20171927

[B56] Laubinger S. Marchal V. Le Gourrierec J. Wenkel S. Adrian J. Jang S. (2006). *Arabidopsis* SPA proteins regulate photoperiodic flowering and interact with the floral inducer CONSTANS to regulate its stability. *Development* 133 3213–3222. 10.1242/dev.02481 16854975

[B57] Lee J. Kang M. H. Kim J. Y. Lim P. O. (2021). The role of light and circadian clock in regulation of leaf senescence. *Front. Plant Sci.* 12:669170. 10.3389/fpls.2021.669170 33912212 PMC8075161

[B58] Lei J. Jayaprakasha G. K. Singh J. Uckoo R. Borrego E. J. Finlayson S. (2019). CIRCADIAN CLOCK-ASSOCIATED1 controls resistance to aphids by altering indole glucosinolate production. *Plant Physiol.* 181 1344–1359. 10.1104/pp.19.00676 31527087 PMC6836836

[B59] Liew L. C. Mohan B. S. Prem L. B. (2017). A novel role of the soybean clock gene LUX ARRHYTHMO in male reproductive development. *Sci. Rep.* 7 1–16. 10.1038/s41598-017-10823-y 28878247 PMC5587693

[B60] Loake G. Grant M. (2007). Salicylic acid in plant defence—The players and protagonists. *Curr. Opin. Plant Biol.* 10 466–472. 10.1016/j.pbi.2007.08.008 17904410

[B61] Locke A. M. Slattery R. A. Ort D. R. (2018). Field-grown soybean transcriptome shows diurnal patterns in photosynthesis-related processes. *Plant Direct* 2:e00099. 10.1002/pld3.99 31245700 PMC6508813

[B62] Lopez L. Fasano C. Perrella G. Facella P. (2021). Cryptochromes and the Circadian Clock: The Story of a Very Complex Relationship in a Spinning World. *Genes* 12:672. 10.3390/genes12050672 33946956 PMC8145066

[B63] Lu H. McClung C. R. Zhang C. (2017). Tick Tock: Circadian Regulation of Plant Innate Immunity. *Annu. Rev. Phytopathol.* 55 287–311. 10.1146/annurev-phyto-080516-035451 28590878

[B64] Lu Y. Gehan J. P. Sharkey T. D. (2005). Daylength and circadian effects on starch degradation and maltose metabolism. *Plant Physiol.* 138 2280–2291. 10.1104/pp.105.061903 16055686 PMC1183414

[B65] Males J. Howard G. (2017). Stomatal biology of CAM plants. *Plant Physiol.* 174 550–560. 10.1104/pp.17.00114 28242656 PMC5462028

[B66] Malik N. A. A. Kumar I. S. Nadarajah K. (2020). Elicitor and Receptor Molecules: Orchestrators of Plant Defense and Immunity. *Int. J. Mol. Sci.* 21:963. 10.3390/ijms21030963 32024003 PMC7037962

[B67] Man A. W. C. Xia N. Li H. (2020). Circadian rhythm in adipose tissue: novel antioxidant target for metabolic and cardiovascular diseases. *Antioxidants* 9:968. 10.3390/antiox9100968 33050331 PMC7601443

[B68] Más P. Alabadí D. Yanovsky M. J. Oyama T. Kay S. A. (2003). Dual role of TOC1 in the control of circadian and photomorphogenic responses in *Arabidopsis*. *Plant Cell* 15 223–236. 10.1105/tpc.006734 12509533 PMC143493

[B69] Matallana-Ramirez L. P. Rauf M. Farage-Barhom S. Dortay H. Xue G. P. Dröge-Laser W. (2013). NAC transcription factor ORE1 and senescence induced bifunctional nuclease1 (BFN1) constitute a regulatory cascade in *Arabidopsis*. *Mol. Plant* 6:1438e1452. 10.1093/mp/sst012 23340744

[B70] McClung C. (2006). Robertson. Plant circadian rhythms. *Plant Cell* 18 792–803. 16595397 10.1105/tpc.106.040980 PMC1425852

[B71] McClung C. R. (2019). The plant circadian oscillator. *Biology* 8:14. 10.3390/biology8010014 30870980 PMC6466001

[B72] McWatters H. G. Kolmos E. Hall A. Doyle M. R. Amasino R. M. Gyula P. (2007). ELF4 is required for oscillatory properties of the circadian clock. *Plant Physiol.* 144 391–401. 10.1104/pp.107.096206 17384164 PMC1913775

[B73] Miller R. N. G. Alves G. S. C. Van Sluys M. A. (2017). Plant immunity: Unravelling the complexity of plant responses to biotic stresses. *Ann. Bot.* 119 681–687. 10.1093/aob/mcw284 28375427 PMC5378191

[B74] Min N. I. (2005). Integration of light signaling with photoperiodic flowering and circadian rhythm. *Cell Res.* 15 559–566. 10.1038/sj.cr.7290325 16117845

[B75] Mizoguchi T. Wright L. Fujiwara S. Cremer F. Lee K. Onouchi H. (2005). Distinct roles of GIGANTEA in promoting flowering and regulating circadian rhythms in *Arabidopsis*. *Plant Cell* 17 2255–2270. 10.1105/tpc.105.033464 16006578 PMC1182487

[B76] Müller L. M. von Korff M. Davis S. J. (2014). Connections between circadian clocks and carbon metabolism reveal species-specific effects on growth control. *J. Exp. Bot.* 65 2915–2923. 10.1093/jxb/eru117 24706717

[B77] Nakamichi N. Kiba T. Henriques R. Mizuno T. Chua N. H. Sakakibara H. (2010). PSEUDO-RESPONSE REGULATORS 9, 7, and 5 are transcriptional repressors in the *Arabidopsis* circadian clock. *Plant Cell* 22 594–605. 10.1105/tpc.109.072892 20233950 PMC2861452

[B78] Nakamichi N. Kita M. Ito S. Yamashino T. Mizuno T. (2005). PSEUDO-RESPONSE REGULATORS, PRR9, PRR7 and PRR5, together play essential roles close to the circadian clock of *Arabidopsis thaliana*. *Plant Cell Physiol.* 46 686–698. 10.1093/pcp/pci086 15767265

[B79] Nassoury N. Fritz L. Morse D. (2001). Circadian changes in ribulose-1,5-bisphosphate carboxylase/oxygenase distribution inside individual chloroplasts can account for the rhythm in dinoflagellate carbon fixation. *Plant Cell* 13 923–934. 10.1105/tpc.13.4.923 11283345 PMC135545

[B80] Nitschke S. Cortleven A. Iven T. Feussner I. Havaux M. Riefler M. (2016). Circadian stress regimes affect the circadian clock and cause jasmonic acid-dependent cell death in cytokinin-deficient *Arabidopsis* plants. *Plant Cell* 28 1616–1639. 10.1105/tpc.16.00016 27354555 PMC4981127

[B81] Nohales M. A. Kay S. A. (2016). Molecular mechanisms at the core of the plant circadian oscillator. *Nat. Struct. Mol. Biol.* 23:1061. 10.1038/nsmb.3327 27922614 PMC7750160

[B82] Pan W. J. Wang X. Deng Y. R. Li J. H. Chen W. Chiang J. Y. (2015). Nondestructive and intuitive determination of circadian chlorophyll rhythms in soybean leaves using multispectral imaging. *Sci. Rep.* 5 1–13. 10.1038/srep11108 26059057 PMC4461922

[B83] Panter P. E. Muranaka T. Cuitun-Coronado D. Graham C. A. Yochikawa A. Kudoh H. (2019). Circadian regulation of the plant transcriptome under natural conditions. *Front. Genet.* 10:1239. 10.3389/fgene.2019.01239 31850080 PMC6895068

[B84] Pokhilko A. Ebenhoh O. (2015). Mathematical modelling of diurnal regulation of carbohydrate allocation by osmo-related processes in plants. *J. R. Soc. Interface* 12 2014–1357. 10.1098/rsif.2014.1357 25631572 PMC4345503

[B85] Rascher U. Hütt M. T. Siebke K. Osmond B. Beck F. Lüttge U. (2001). Spatiotemporal variation of metabolism in a plant circadian rhythm: the biological clock as an assembly of coupled individual oscillators. *Proc. Nat. Acad. Sci. U S A.* 98 11801–11805. 10.1073/pnas.191169598 11573013 PMC58811

[B86] Rawat R. Takahashi N. Hsu P. Y. Matthew A. Jacob Schwartz J. Salemi M. R. (2011). REVEILLE8 and PSEUDO-REPONSE REGULATOR5 form a negative feedback loop within the *Arabidopsis* circadian clock. *PLoS Genet.* 7:e1001350. 10.1371/journal.pgen.1001350 21483796 PMC3069099

[B87] Roden L. C. Ingle R. A. (2009). Lights, rhythms, infection: the role of light and the circadian clock in determining the outcome of plant–pathogen interactions. *Plant Cell* 21 2546–2552. 10.1105/tpc.109.069922 19789275 PMC2768925

[B88] Rugnone M. L. Soverna A. F. Sanchez S. E. Schlaen R. G. Hernando C. E. Seymour D. K. (2013). LNK genes integrate light and clock signaling networks at the core of the *Arabidopsis* oscillator. *Proc. Nat. Acad. Sci. U S A.* 110 12120–12125. 10.1073/pnas.1302170110 23818596 PMC3718124

[B89] Saini R. Jaskolski M. Davis S. J. (2019). Circadian oscillator proteins across the kingdoms of life: structural aspects. *BMC Biol.* 17 1–39. 10.1186/s12915-018-0623-3 30777051 PMC6378743

[B90] Sanchez A. Shin J. Davis S. J. (2011). Abiotic stress and the plant circadian clock. *Plant Signal. Behav.* 6 223–231. 10.4161/psb.6.2.14893 21325898 PMC3121982

[B91] Sawa M. Kay S. A. (2011). GIGANTEA directly activates FLOWERING LOCUS T in Arabidopsis thaliana. *Proc. Nat. Acad. Sci. U S A.* 108 11698–11703. 10.1073/pnas.1106771108 21709243 PMC3136272

[B92] Sawa M. Nusinow D. A. Kay S. A. Imaizumi T. (2007). FKF1 and GIGANTEA complex formation is required for day-length measurement in Arabidopsis. *Science* 318 261–265. 10.1126/science.1146994 17872410 PMC3709017

[B93] Schaffer R. Ramsay N. Samach A. Corden S. Putterill J. Carré I. A. (1998). The late elongated hypocotyl mutation of *Arabidopsis* disrupts circadian rhythms and the photoperiodic control of flowering. *Cell* 1998 1219–1229. 10.1016/s0092-8674(00)81465-8 9657154

[B94] Shogo I. Niwa Y. Nakamichi N. Kawamura H. Yamashino T. Mizuno T. (2008). Insight into missing genetic links between two evening-expressed pseudo-response regulator genes *TOC1* and *PRR5* in the circadian clock-controlled circuitry in *Arabidopsis thaliana*. *Plant Cell Physiol.* 49 201–213. 10.1093/pcp/pcm178 18178585

[B95] Shor E. Paik I. Kangisser S. Green R. Huq E. (2017). PHYTOCHROME INTERACTING FACTORS mediate metabolic control of the circadian system in *Arabidopsis*. *New Phytol.* 215 217–228. 10.1111/nph.14579 28440582 PMC5458605

[B96] Smith A. M. Stitt M. (2007). Coordination of carbon supply and plant growth. *Plant Cell Environ.* 30 1126–1149. 10.1111/j.1365-3040.2007.01708.x 17661751

[B97] Song Y. H. Smith R. W. To B. J. Millar A. J. Imaizumi T. (2012). FKF1 conveys timing information for CONSTANS stabilization in photoperiodic flowering. *Science* 336 1045–1049. 10.1126/science.1219644 22628657 PMC3737243

[B98] Spoel S. H. Dong X. (2008). Making Sense of Hormone Crosstalk during Plant Immune Responses. *Cell Host Microbe* 3 348–351. 10.1016/j.chom.2008.05.009 18541211

[B99] Srivastava D. Shamim M. D. Kumar M. Mishra A. Maurya R. Sharma D. (2019). Role of circadian rhythm in plant system: An update from development to stress response. *Environ. Exp. Bot.* 162 256–271. 10.1186/s13054-016-1208-6 27885969 PMC5493079

[B100] Stitt M. Zeeman S. C. (2012). Starch turnover: Pathways, regulation and role in growth. *Curr. Opin. Plant Biol.* 15 282–292. 10.1016/j.pbi.2012.03.016 22541711

[B101] Suárez-López P. Wheatley K. Robson F. Onouchi H. Valverde F. Coupland G. (2001). CONSTANS mediates between the circadian clock and the control of flowering in Arabidopsis. *Nature* 410 1116–1120. 10.1038/35074138 11323677

[B102] Sulpice R. Flis A. Ivakov A. A. Apelt F. Krohn N. Encke B. (2014). *Arabidopsis* coordinates the diurnal regulation of carbon allocation and growth across a wide range of photoperiods. *Mol. Plant* 7 137–155. 10.1093/mp/sst127 24121291

[B103] Tamaoki D. Seo S. Yamada S. Kano A. Miyamoto A. Shishido H. (2013). Jasmonic acid and salicylic acid activate a common defense system in rice. *Plant Signal. Behav.* 8:e24260. 10.4161/psb.24260 23518581 PMC3906320

[B104] Thines B. Katsir L. Melotto M. Niu Y. Mandaokar A. Liu G. (2007). JAZ repressor proteins are targets of the SCF(COI1) complex during jasmonate signalling. *Nature* 448 661–665. 10.1038/nature05960 17637677

[B105] Valverde F. Mouradov A. Soppe W. Ravenscroft D. Samach A. Coupland G. (2004). Photoreceptor regulation of CONSTANS protein in photoperiodic flowering. *Science* 303 1003–1006. 10.1126/science.1091761 14963328

[B106] Wang X. Ma L. (2013). Unraveling the circadian clock in *Arabidopsis*. *Plant Signal Behav.* 8:e23014. 10.4161/psb.23014 23221775 PMC3657000

[B107] Wasternack C. Hause B. (2013). Jasmonates: biosynthesis, perception, signal transduction and action in plant stress response, growth and development. An update to the 2007 review in *Annals of Botany*. *Ann. Bot.* 111 1021–1058. 10.1093/aob/mct067 23558912 PMC3662512

[B108] Webb A. A. R. (2003). The physiology of circadian rhythms in plants. *New Phytol.* 160 281–303. 10.1046/j.1469-8137.2003.00895.x 33832173

[B109] Wu J. F. Tsai H. L. Joanito I. Wu Y. C. Chang C. E. Li Y. H. (2016). LWD–TCP complex activates the morning gene CCA1 in *Arabidopsis*. *Nat. Commun.* 7 1–10. 10.1038/ncomms13181 27734958 PMC5065627

[B110] Yan J. Kim Y. J. Somers D. E. (2021). Post-Translational Mechanisms of Plant Circadian Regulation. *Genes* 12:325. 10.3390/genes12030325 33668215 PMC7995963

[B111] Yarkhunova Y. Edwards C. E. Ewers B. E. Baker R. L. Aston T. L. McClung C. R. (2016). Selection during crop diversification involves correlated evolution of the circadian clock and ecophysiological traits in *Brassica rapa*. *New Phytol.* 210 133–144. 10.1111/nph.13758 26618783

[B112] Yazdanbakhsh N. Sulpice R. Graf A. Stitt M. Fisahn J. (2011). Circadian control of root elongation and C partitioning in *Arabidopsis* thaliana. *Plant Cell Environ.* 34 877–894. 10.1111/j.1365-3040.2011.02286.x 21332506

[B113] Yeom M. Kim H. Lim J. Shin A. Y. Hong S. Kim J. I. (2014). How do phytochromes transmit the light quality information to the circadian clock in *Arabidopsis*? *Mol. Plant* 7 1701–1704. 10.1093/mp/ssu086 25095795

[B114] Youngsung J. Fragoso V. Yon F. Baldwin I. T. Kim S. G. (2017). Circadian clock component, LHY, tells a plant when to respond photosynthetically to light in nature. *J. Int. Plant Biol.* 59 572–587. 10.1111/jipb.12547 28429400

[B115] Yuan L. Yu Y. Liu M. Song Y. Li H. Sun J. (2021). BBX19 fine-tunes the circadian rhythm by interacting with PSEUDO-RESPONSE REGULATOR proteins to facilitate their repressive effect on morning-phased clock genes. *Plant Cell.* 33 2602–2617. 10.1093/plcell/koab133 34164694 PMC8408442

[B116] Zhang C. Min G. Seitz N. C. Angel W. Hallworth A. Wiratan L. (2019). LUX ARRHYTHMO mediates crosstalk between the circadian clock and defense in *Arabidopsis*. *Nat. Commun.* 10 1–14. 10.1038/s41467-019-10485-6 31186426 PMC6560066

[B117] Zhang J. Ren Z. Zhou Y. Ma Z. Ma Y. Hou D. (2019). NPR1 and Redox Rhythms: Connections, between Circadian Clock and Plant Immunity. *Int. J. Mol. Sci.* 20:1211. 10.3390/ijms20051211 30857376 PMC6429127

[B118] Zhang Y. Wang Y. Wei H. Li N. Tian W. Chong K. (2018). Circadian evening complex represses jasmonate-induced leaf senescence in *Arabidopsis*. *Mol. Plant* 11 326–337. 10.1016/j.molp.2017.12.017 29306046

[B119] Zuo Z. Liu H. Liu B. Liu X. Lin C. (2011). Blue light dependent interaction of CRY2 with SPA1 regulates COP1 activity and floral initiation in *Arabidopsis*. *Curr. Biol.* 21 841–847. 10.1016/j.cub.2011.03.048 21514160 PMC3150455

